# On the biological relevance of MHC class II and B7 expression by tumour cells in melanoma metastases

**DOI:** 10.1038/sj.bjc.6600703

**Published:** 2003-02-10

**Authors:** M R Bernsen, L Håkansson, B Gustafsson, L Krysander, B Rettrup, D Ruiter, A Håkansson

**Affiliations:** 1 Division of Clinical Tumour Immunology, Department of Oncology, University Hospital, Linköping, Sweden; 2 Department of Pathology and Cytology, University Hospital, Linköping, Sweden; 3 Department of Hand and Plastic Surgery, University Hospital, Linköping; 4 Department of Surgery, County Hospital, Kalmar, Sweden; 5 Department of Pathology, University Medical Center St. Radboud, Nijmegen, The Netherlands

**Keywords:** MHC II, melanoma, biochemotherapy, B7.1, B7.2, response

## Abstract

A large number of studies have indicated that specific immune reactivity plays a crucial role in the control of malignant melanoma. In this context, expression of MHC I, MHC II and B7 molecules by melanoma cells is seen as relevant for the immune response against the tumour. For a better understanding of the biological relevance of MHC II and B7 expression by tumour cells in metastatic melanoma, we studied the expression of these molecules in melanoma metastases in relation to the inflammatory response, regression of the tumour and survival from 27 patients treated with biochemotherapy (30 mg m^−2^ Cisplatin and 250 mg m^−2^ decarbazine (dimethyl-triazene-imidazole-carboxamide, DTIC) on days 1–3 i.v., and 10^7^ IU IFN-*α*2b 3 days a week s.c., q. 28d). In 19 out of 27 lesions studied, we found expression of MHC II by the tumour cells, while only in one out of 11 tumour biopsies obtained from untreated metastatic melanoma patients, MHC II expression was detected. Expression of B7.1 and B7.2 by tumour cells was found in nine out of 24 and 19 out of 24 lesions, respectively. In all cases where B7.1 expression was found, expression of B7.2 by the tumour cells was also seen. In general, no or only few inflammatory cells positive for B7 were found. Expression of MHC II by tumour cells was positively correlated with the presence of tumour-infiltrating lymphocytes, regression of the lesion, and with time to progression (TTP) and overall survival (OS) of the patient. However, no significant correlation between B7.1 or B7.2 expression and regression of the tumour, TTP or OS was found. In light of other recent findings, these data altogether do support a role as biomarker for MHC II expression by tumour cells; however, its exact immunological pathomechanism(s) remain to be established.

Many studies have indicated that the immune system is capable of controlling tumour growth in cancer patients, especially in melanoma patients. Supportive findings comprise the occurrence of spontaneous regressions in primary melanomas (McGovern, 1975; Kang *et al*, 1993), the prognostic relevance of the presence of an inflammatory infiltrate in melanomas (Clemente *et al*, 1996; Mihm *et al*, 1996), the selective reactivity of tumour-infiltrating lymphocytes (TIL) against autologous tumour cells in 50% of patients with metastatic melanoma (Castelli *et al*, 1997), and the occurrence of objective responses in cancer patients following immune-modulating treatment (Mukherji and Chakraborty, 1995; Davis, 2000). From studies in animal tumour models and some clinical observations, it has been shown that specific T lymphocytes fulfil a crucial role in the immunological control of tumour growth (Castelli *et al*, 1997; Coulie *et al*, 1999; Yamshchikov *et al*, 2001). For T-cell activation and recognition of tumour cells by T cells, antigen presentation in the context of MHC molecules is required. The specific trigger for CD8+ and CD4+ T cells consists of antigenic peptides complexed with MHC class I molecules or MHC class II molecules, respectively. Expression of these antigens by tumour cells is therefore considered relevant for the immunological control of tumour growth. MHC I is normally expressed by all nucleated cells. Data on the expression of MHC I by tumour cells are consistent in that expression of these molecules appears to be crucial for effective immune recognition and consequently for immunological control of tumour growth. Loss of MHC I expression is considered as one of the major immune escape mechanisms exploited by tumour cells (Ruiter *et al*, 1982; Marincola *et al*, 1992; Rees and Mian, 1999; Algarra *et al*, 2000).

MHC II is normally not expressed by nonprofessional antigen-presenting cells (APC). Data on the relevance of MHC II expression by tumour cells for immunological control of tumour growth is, in contrast to data on MHC I, not consistent. Melanocytes in normal skin and common nevi are negative for MHC class II (Guerry *et al*, 1987; Ruiter *et al*, 1991), while MHC class II expression on melanocytes does appear with neoplastic transformation (Guerry *et al*, 1987; Ruiter *et al*, 1991) and may increase with increasing malignancy or lesion thickness (Bröcker *et al*, 1984; Becker *et al*, 1993). IFN-*γ* induces MHC class II expression in melanoma cells as well as in melanocytes (Guerry *et al*, 1987). In a number of mouse tumour models, tumour cells transfected with MHC class II show an increased immunogenicity compared to the parental cell line. These cell lines also show CD4+ T cell stimulation *in vitro*. It is hypothesised that the transfected tumour cells act as direct APC to CD4+ cells, presenting endogenous tumour antigens (Chen *et al*, 1994; Pulaski and Ostrand-Rosenberg, 1998; Ostrand-Rosenberg *et al*, 1999). In human primary melanoma, however, expression of MHC class II by tumour cells is associated with an unfavourable prognosis (van Duinen *et al*, 1988; Ruiter *et al*, 1991). In addition, Becker *et al* (1993) reported that autologous melanoma cell lines render CD4+ T cells unresponsive to subsequent stimulation in an MHC class II restricted manner. This effect could be abrogated by transfection of the melanoma cells with the costimulatory molecule B7 (Becker *et al*, 1993). In summary, while studies in some animal tumour models suggest that MHC II expression by tumour cells increases the immunogenicity of the tumour, data on human tumour cells suggest that MHC II expression might reduce the immunogenicity of the tumour in patients with melanoma metastases.

In this paper, we address the relevance of expression of MHC II and the costimulatory molecules B7.1 and B7.2 in relation to response to treatment with biochemotherapy.

## MATERIALS AND METHODS

### Patient data

The present study describes 27 patients (18 males and nine females) with metastatic malignant melanoma, 15 with regional disease and 12 with resectable systemic disease. The median age was 58 (range 34–71) years and their Karnofsky status was 70 or more. Recurrences were cytologically verified by fine-needle aspirates before treatment was started. One metastasis from each patient was studied. In patients with systemic disease, easily resectable metastases were chosen. In patients with regional disease, non-necrotic biopsies were randomly chosen by the pathologist. In addition, tumour biopsies obtained from 11 untreated metastatic melanoma patients (nine males, two females) were studied for MHC II expression. All these patients had regional disease and a median age of 57 (range 39–79) years. Recurrences were cytologically verified by fine-needle aspirates. One metastasis from each patient was studied.

The study was approved by the ethical committee at the University Hospital of Linköping, Sweden.

### Pretreatment investigations and treatment schedule

Pretreatment investigations were as described previously (Håkansson *et al*, 2001). Treatment consisted of 30 mg m^−2^ cisplatin (*cis*-diamine-dichloro-platinum) and 250 mg m^−2^ dacarbazine (dimethyl-triazene-imidazole-carboxamide, DTIC) on days 1–3 i.v., and 10^7^ IU IFN-*α*2b 3 days a week s.c. The duration of one cycle was 28 days.

### Preparation of tumour biopsies and immunological staining of tissue sections

Biopsies from the resected metastases were immediately snap frozen and stored at −70°C until further use. Tissue sections, 6–7 *μ*m thick, were fixed with phosphate-buffered 4% paraformaldehyde, pH 7.4 (Riedel-de Haen Ag, Seelze, Germany) supplemented with 5.4 g l^−1^ of glucose for 5 min and then washed three times in Hanks' balanced salt solution (BSS, Gibco, Paisley, UK), supplemented with 0.01 M Hepes solution before staining. For detection of the expression of MHC antigens, B7.1, B7.2 and the presence of T-cell subsets, the following monoclonal antibodies were used: anti-HLA, Stockholm, Sweden, DR for MHC II (0.5 *μ*g ml^−1^, clone L243; Becton Dickinson), anti-HLA-ABC for MHC I (0.57 *μ*g ml^−1^, clone W6/32; Dakopatts, Oxon, UK), anti-B7.1 (20 *μ*g ml^−1^, clone 37711.11; R&D systems), anti-B7.2 (20 *μ*g ml^−1^, clone 37301.11; R&D systems), anti-CD3 (7.5 *μ*g ml^−1^, clone UCHT1: Dako), leu-3a for CD4 (1 *μ*g ml^−1^; Becton Dickinson) and Leu-2a for CD8 (0.25 *μ*g ml^−1^; Becton Dickinson). Sections for T-cell subsets were blocked with normal rabbit serum before the first staining. Sections for MHC I and MHC II were blocked with normal goat serum and sections for B7.1 and B7.2 with 10% AB serum. Tissue sections were then incubated with primary antibodies for 30 min or overnight (B7.1 and B7.2) at room temperature. Mouse IgG1 (Dakopatts, Sweden) was used as a negative control for the subsets of inflammatory cells and the B7 antibodies. Mouse IgG (Sigma, Stockholm, Sweden) was used as a negative control for the major histocompatibility antigens. After the slides were washed in BSS-saponin, biotinylated rabbit-anti-mouse immunoglobulin was added at a 1 : 100 dilution in BSS-saponin for anti-CD3, anti-CD4 and anti-CD8. They were then incubated with peroxidase-labelled streptavidin (P0397, DAKO, Stockholm, Sweden) at a 1 : 100 dilution in BSS-saponin for 30 min. DAB (3,3′-diaminobenzidine, D-5637, Sigma, Stockholm, Sweden) was used as a substrate. Sections stained for MHC I and MHC II were washed in BSS-saponin; then, goat-anti-mouse immunoglobulin was added at a 1 : 25 dilution in BSS-saponin and left to incubate for 30 min in a moist chamber. The slides were again washed in BSS-saponin, and mouse APAAP complex diluted at 1 : 25 in BSS-saponin was added and incubated for 30 min. For B7.1 and B7.2, the Envision system was used as the second step (DAKO, Stockholm, Sweden). All antibody solutions contained 2% normal blood donor AB serum. After washes in BSS-saponin and TBS, the Fast-red substrate supplemented with levamisole to block endogenous alkaline phosphatase was added to the sections (MHC and B7) and left to incubate for 20 min at room temperature. The slides were then washed in TBS, pH 7.6, counterstained in Mayer's haematoxylin for 15 min and mounted in Glycergel (Dakopatts, Sweden).

### Evaluation of tumour regression and histopathological criteria

The occurrence of tumour regression was evaluated by histopath-ological examination of tumour biopsies based on the description of regressive changes in malignant melanoma as previously described (McGovern, 1975; Kang *et al*, 1993; Håkansson *et al*, 1996,^1998^). The following criteria of tumour regression were used in this study: (1) low and variable density of tumour cells, particularly variation in density within the same tumour nodule; (2) disorganisation of the architecture of the tumour with nests of remaining tumour cells surrounded by stromal tissue and (3) fibrosis. The mononuclear infiltrate was not used as a criterion of histopathological tumour regression in this study. The regressive changes were scored on control sections not stained for mononuclear cells. The signs of regression vary from no signs to almost complete destruction with only a few tumour cells present. The degree of tumour regression was scored semiquantitatively as less than 10%, comprising 10–25, 25–50, 50–75 or 75–100% of the section area. A score of >25% was generally considered significant unless otherwise stated. The scoring was performed independently by two observers (MRB, BG) and consensus was reached for all scorings.

### Statistical analysis

Survival curves were plotted using the Kaplan–Meier method. Differences in clinical response in terms of time to progression and OS between patients showing differences in expression of MHC II or B7 by the tumour or differences in regressive changes in the tumour were analysed using the log rank test. A two-sided Fisher's exact test was used to analyse whether or not an increased level of MHC II expression coincided with an increased level of histological regression. Calculations of significance were performed using Graph Pad Prism version 3.02 for Windows (Graph Pad Software, San Diego, CA, USA).

## RESULTS

### MHC I and MHC II expression in metastatic lesions from treated and untreated melanoma patients

**Figure 1:**
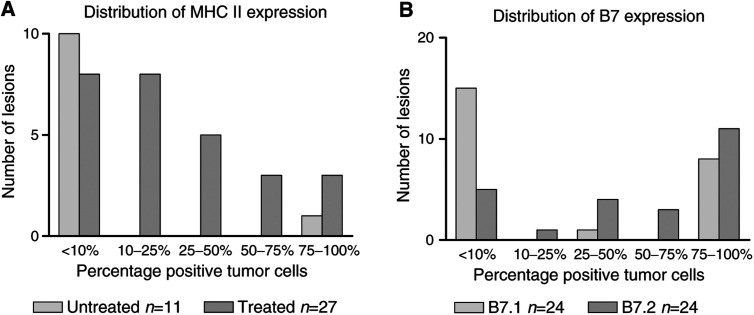
Distribution of MHC II (**A**) and B7 (**B**) expression over the lesions studied.

**Figure 2:**
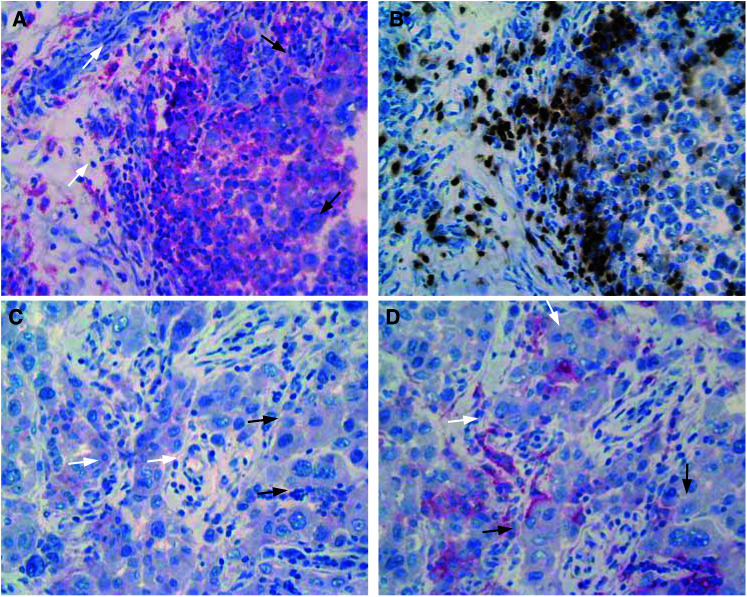
Expression of MHC II (**A**: red-stained cells, black arrows indicating positive tumour cells and white arrows indicating positive inflammatory cells) and presence of CD3+ cells (**B**: brown-stained cells; the brown staining is not melanin) in corresponding areas of a metastatic lesion. Expression of B7.1 (**C**: black arrows indicating positive-staining tumour cells and white arrows indicating negative inflammatory cells) and B7.2 (**D**: black arrows indicating positive tumour cells and white arrows indicating positive inflammatory cells) in corresponding areas of a metastatic lesion (red-stained cells).

The expression of MHC classes I and II was studied in 27 lesions from biochemotherapy-treated patients by immunohistochemical analysis. All lesions were homogeneously positive for MHC I. In contrast, a heterogeneous expression of MHC II was found between and within lesions. In eight out of these 27 lesions, the tumour cells were negative for MHC II, while in the remaining 19 lesions, 10 to more than 75% of the tumour cells were positive for MHC II (Figure 1A

). In contrast, in 11 tumour biopsies derived from untreated patients, only in one out of the 11 MHC II expression was detected in approximately 75% of the tumour cells. In all cases, MHC II expression of the tumour cells was associated with a pronounced presence of TIL as exemplified in Figure 2A and B

. In all cases, these TIL consisted of CD4+ as well as CD8+ cells. There was some heterogeneity in the relative occurrence of these subpopulations, but this heterogeneity could not be linked to whether or not adjacent tumour cells were positive for MHC II. In all cases, the majority of inflammatory cells were MHC II-positive.

### B7 expression in metastatic lesions of treated melanoma patients

**Figure 3:**
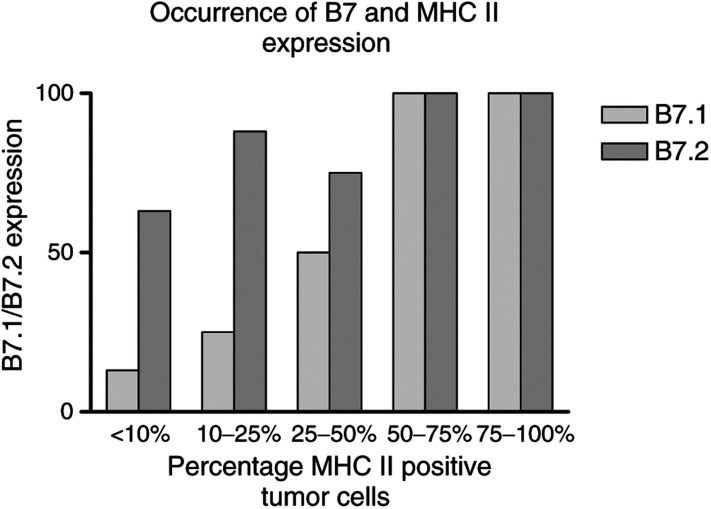
Relative distribution of B7.1 and B7.2 expression in relation to MHC II expression.

Also for B7, heterogeneous expression between and within lesions was found. Out of 24 lesions studied for B7.1 and B7.2 expression, 15 were negative for B7.1 and five for B7.2 (Figure 1B). In most cases where B7 expression was detected, the staining intensity of the tumour cells was weak as compared to the staining intensity of inflammatory cells when positive (Figure 2C). For B7.1 in none of the 24 lesions, positive-staining inflammatory cells were observed. In contrast, for B7.2 in 17 out of 24 lesions, positive staining of inflammatory cells was seen (Figure 2C and D). In general, however, only a limited fraction of the inflammatory cells were positive for B7.2. In all lesions where B7.1 positive tumour cells were found, positive staining for B7.2 was also found. In Figure 3

the relative distribution of B7.1 and B7.2 positive tumours, that is more than 10% of the tumour cells positive, in relation to the expression of MHC II by tumour cells is shown. Although it seems that B7.1 expression is found more frequently in tumours that are significantly positive for MHC II, that is more than 25% of the tumour cells positive for MHC II, no specific relation between MHC II expression and B7.1 or B7.2 expression could be discerned.

### Relation between MHC II and B7 expression by tumour cells and the occurrence of histological regression in metastatic lesions of melanoma patients

**Figure 4:**
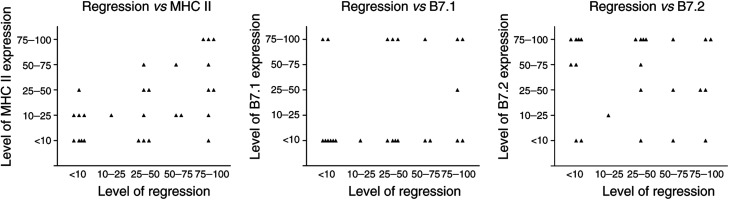
Relation between the extent of regression and expression of MHC II (**A**), B7.1 (**B**) and B7.2 (**C**).

Histological regression of tumour lesions was scored according to criteria as published before (McGovern, 1975; Kang *et al*, 1993; Håkansson *et al*, 1996,^1998^) and as specified under Materials and Methods. We plotted the extent of histological regression against the percentage of MHC II-positive tumour cells found in each lesion (Figure 4A

). In 18 out of 27 lesions studied, regressive changes were observed. Out of these 18, 10 were significantly positive for MHC II, that is more than 25% of the tumour cells, while in the remaining nine lesions that showed no significant regressive changes, that is less than 25% of the section area, only in one out of nine lesions MHC II expression in more than 25% of the tumour cells was detected. This difference was statistically significant (*P*=0.042; two-sided Fisher's exact test).

Similar to MHC II, we plotted the extent of histological regression against the percentage of B7-positive tumour cells found in each lesion (Figure 4B and C). There was, however, no apparent relation between the occurrence of regressive changes and B7 expression by the tumour cells.

### Relevance of MHC II and B7 expression by tumour cells to clinical response parameters

**Figure 5:**
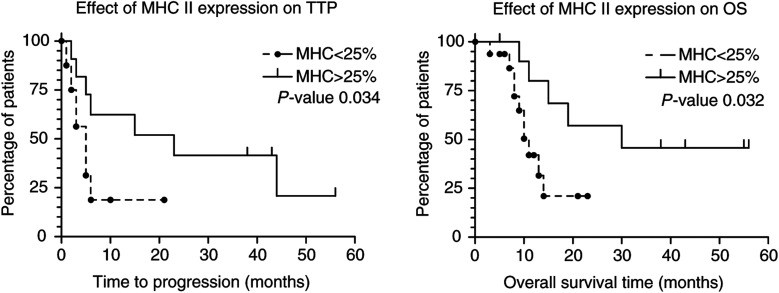
Kaplan–Meier analysis for TTP and OS on patients whose tumour showed significant levels, that is >25% of the tumour cells, of MHC II expression.

In order to establish whether or not expression of MHC II and or B7 by tumour cells might be of clinical relevance, we performed survival analysis on patients in relation to the extent to which their tumour expressed MHC II or B7. For TTP as well as OS, there was a significant trend for increased survival in relation to increased expression of MHC II by the tumour (*P*=0.034 and 0.043, respectively; log rank test for trend). The use of a cutoff point of 25% of the tumour cells positive for MHC II results in a significant difference in TTP and OS for the two resulting patient groups (*P*=0.034 and 0.032, respectively; Figure 5

).

**Figure 6:**
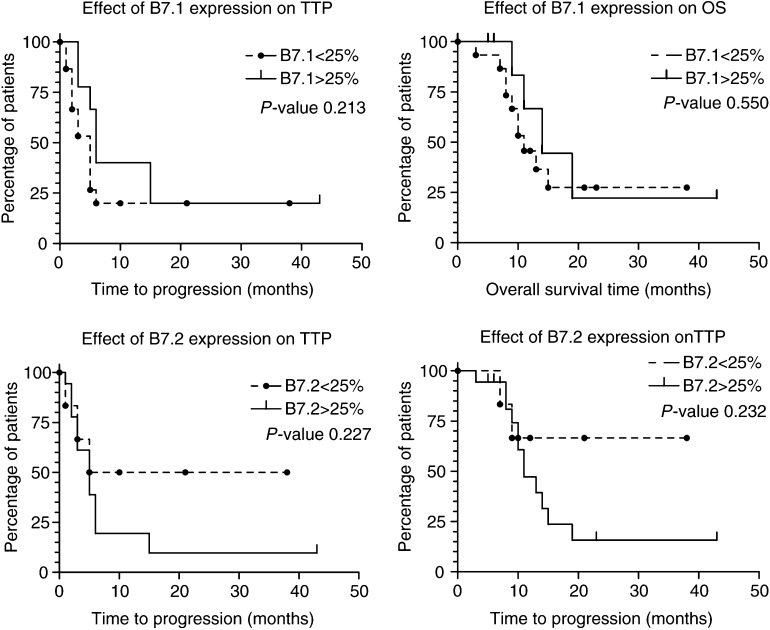
Kaplan–Meier analysis for TTP and OS on patients whose tumour showed different levels of B7.1 and B7.2 expression.

A similar approach was used for the relation between B7 expression by the tumour and TTP and OS of the patients. No significant relation between B7 expression by the tumour and TTP or OS was found (Figure 6

).

## DISCUSSION

Metastatic malignant melanoma is, despite various treatment strategies, still associated with a poor prognosis. In other studies, we have demonstrated that treatment with IFN*α* or IFN*α* in combination with cisplatin and DTIC results in early (incomplete) regression of metastases (Håkansson *et al*, 1996,^2001^). These responses showed a strong correlation with the amount of tumour-infiltrating CD4+ lymphocytes present before treatment. However, objective remission following IFN*α*-based treatment is still only 15% (Legha, 1997). These observations suggest that an initial effective antitumour immune response is initiated following treatment, but that shortly thereafter immunological resistance occurs (Håkansson *et al*, 1999,^2001^).

Given the apparent important role of CD4+ cells in the induction of an effective immune response to the tumour, we studied the expression of MHC II and B7 in melanoma metastases of patients treated with IFN*α*, cisplatin and DTIC, in relation to the inflammatory response, regression of the tumour and survival of the patients. In 19 out of 27 lesions studied, we found expression of MHC II by the tumour cells. In contrast, in 11 tumour biopsies obtained from metastatic melanoma patients before treatment, only in one lesion MHC II expression by the tumour cells was seen. These findings suggest a treatment-related expression of MHC II by the tumour. This is further supported by the findings in three fine-needle aspirate biopsies derived from three patients before treatment (data not shown). In none of the three aspirates positive staining of the tumour cells for MHC II was obtained, while in two of the three corresponding post-treatment tumour biopsies MHC II-positive tumour cells were detected (25–50 and 75–100%, respectively). As also found by others (Bröcker *et al*, 1984,^1988^; Guerry *et al*, 1987; Hersey and Jamal, 1990), we found a close correlation between MHC II expression and presence of TIL. There was also a positive correlation between MHC II expression by tumour cells and regression of the lesion. MHC II expression was also positively correlated with TTP and OS of the patients. Patients whose tumours showed MHC II-positive staining of more than 25% of the tumour cells had a significant longer TTP and OS survival than those whose tumour was negative or only limited positive (less than 25% of the tumour cells) for MHC II. In this study, MHC II expression on tumour cells thus represents a biomarker for response. This expression of MHC II by tumour cells might be of particular importance with regard to the observation that the occurrence of CD4+ lymphocytes before initiation of IFN-*α* treatment or biochemotherapy was significantly correlated to therapeutic response (Håkansson *et al*, 1996,^2001^). During IFN-*α* treatment, these lymphocytes were also shown to migrate from the stromal areas surrounding the tumour nodules into these nodules close to the tumour cells. Related to this redistribution of the lymphocytes regressive tumour changes appeared. These findings are highly suggestive of an antitumour activity exerted by CD4+ lymphocytes. A prerequisite for an antitumour activity by these lymphocytes is, however, that the tumour cells express MHC II (Zennadi *et al*, 2001).

It is conceivable that MHC II expression by the tumour cells is a result of inflammatory regression of the tumour following treatment. In this inflammatory milieu IFN*γ* most likely will be produced, resulting in the local induction of MHC II expression by tumour cells, which would be in accordance with the data presented in this paper. However, it remains arguable whether the MHC II-positive tumour cells have a local stimulatory effect on tumour infiltrating CD4+ cells or a suppressive effect or no effect at all. *In vitro* studies have shown that binding of CD4+ cells to MHC II in the absence of B7 molecules can lead to an immune suppressed or anergic state of the CD4+ cell (Becker *et al*, 1993). MHC II expression by nonprofessional APC is also considered a negative feedback mechanism to maintain peripheral tolerance (Marelli-Berg and Lechler, 1999). In this light, MHC II expression by tumour cells could represent a negative factor in the immunological control of cancer and may be an explanation for the finding that MHC II expression in primary melanoma and locoregional melanoma metastases is associated with an unfavourable prognosis (van Duinen *et al*, 1988; Ruiter *et al*, 1991). In the present study, however, MHC II expression by tumour cells in metastatic lesions from biochemotherapy-treated patients was associated with longer TTP and OS. As shown in this study, in the majority of MHC II-positive lesions also expression of B7.1 and/or B7.2 by tumour cells was found. Possibly, this coexpression of MHC II and B7 increases the immunogenicity of the tumour as has been demonstrated in *in vitro* studies and mouse tumour models (Becker *et al*, 1993; Chen *et al*, 1994; Baskar *et al*, 1996; Pulaski and Ostrand-Rosenberg, 1998; Ostrand-Rosenberg *et al*, 1999). Surprisingly, however, no significant correlation between B7.1 or B7.2 expression in itself and regression of the tumour, TTP or OS was found. In any case, the question as to why in most patients the immunological control of tumour growth is only partial still remains. In this respect the results presented in this paper could also be seen in a different light. As mentioned earlier, it is conceivable that MHC II expression by the tumour cells, as frequently observed in the metastatic lesions of biochemotherapy-treated patients, is a result of the local inflammatory milieu following immune reactivity against the tumour, thus indicating the successful (systemic) induction of an antitumour immune response, and hence associated with regressive changes in the lesions and longer TTP and OS of the patients. Locally, the expression of MHC II on tumour cells may still have a detrimental effect on the T-cell response by causing local downregulation or anergy of the T cells. Possibly, this might influence the expression of CD28 and the *ξ*-chain, as these molecules were found to be downregulated, in particular, in regressive tumour areas during IFN-*α* treatment or biochemotherapy (Håkansson *et al*, 1999). In this respect, data on the heterogeneous effects of B7 molecules on T-cell modulation may also be relevant (Martin-Fontecha *et al*, 1996; Kosmaczewska *et al*, 2001). Both B7.1 and B7.2 bind to CD28 and CTLA4, but with different affinity and possibly resulting in different signalling pathways (Martin-Fontecha *et al*, 1996; Kosmaczewska *et al*, 2001). For instance, while CD28 triggering has been shown to be crucial in T-cell activation, CTLA4 triggering appears to be mainly associated with downregulation or inhibition of T-cell responses (Greenfield *et al*, 1998; Kosmaczewska *et al*, 2001). Also the role of other costimulatory molecules may be of significance. Although B7 molecules are often seen as crucial factors for costimulation, some reports have also indicated that other costimulatory molecules, for example, ICAM-1, may be sufficient for efficient T-cell activation (Robinson and Delvig, 2002).

Other recent findings, however, may also cast a different light on the immunological significance of MHC II expression by tumour cells. The relevance of tumour cells as APC for CD4+ cells appears to be dependent on the coexpression of MHC II and accessory molecules such as the invariant chain (Ii) and HLA-DM (Armstrong *et al*, 1997; Brocke *et al*, 2002), each of which are under the primary control of CIITA, the Class II transactivator. Normally MHC II is expressed in concert with these accessory molecules and mainly exogenous antigens are presented (Masternak *et al*, 2000; Robinson and Delvig, 2002). In this situation, tumour cells appear to be poor immunogens (Armstrong *et al*, 1997; Blanck, 1999). However, when MHC II is expressed without coexpression of Ii and HLA-DM, such as in MHC II-transduced or -transfected tumour cells, they may become potent immunogens by presenting endogenous antigens, provided that B7 expression is induced following MHC II engagement or expressed through cotransfection or cotransduction of the B7 gene(s) (Baskar *et al*, 1996; Armstrong *et al*, 1997). MHC II-expressing tumour cells in tumours may thus be poor APC or even immunologically inert to CD4+ cells. The question then remains as to why in certain cases MHC II expression by tumour cells is associated with a poor prognosis in melanoma and why in other cases, as in the present study, MHC II expression is positively correlated with clinical response parameters. As mentioned earlier, expression of MHC II and its accessory molecules is primarily controlled by CIITA. For CIITA expression itself, four different promoters have been identified (Muhlethaler-Mottet *et al*, 1997). Each promoter is functional in different cell types and/or under different physiological conditions (Muhlethaler-Mottet *et al*, 1997; van der Stoep *et al*, 2002). Promoters I and III direct specific constitutive expression of CIITA in dendritic cells and B cells, respectively, while promoter IV is inducible by IFN-*γ* and is responsible for the IFN-*γ*-inducible expression of CIITA and thus MHC II in nonprofessional APC. The function of promoter II is still unknown. In melanoma cell lines, for a nonprofessional APC, the uncommon feature of constitutive expression of MHC II has frequently been described (Nistico *et al*, 1990; Goodwin *et al*, 2001). Deffrennes *et al* (2001) recently found that in a number of melanoma cell lines, constitutive expression of MHC II was associated with constitutive expression of CIITA, which was, against their expectations, initiated from promoter III, and propose that this feature is not a random event but is linked to the neoplastic state of melanoma cells. These findings open up the possibility that MHC II expression by tumour cells may be of limited immunological significance and represent an epiphenomenon of other biological processes. If MHC II expression by melanoma cells, as often seen in primary melanomas and locoregional metastases, is because of constitutive expression of CIITA initiated from promoter III as a consequence of dedifferentiation of the tumour cells (Deffrennes *et al*, 2001; van der Stoep *et al*, 2002), the poor prognosis for patients with MHC II-positive tumours may be a result of the more malignant phenotype of the tumour than any effect of the MHC II expression in itself. In contrast, in cases where MHC II expression by tumour cells is because of IFN-*γ*-induced expression of CIITA, the expression of MHC by tumour cells may be linked to an ongoing local antitumour immune response with local IFN-*γ* production, and would therefore be positively correlated to clinical response parameters as already suggested above. Whether the MHC II expression itself is then of immunological significance remains to be established.

In conclusion, MHC II expression in metastatic melanoma is relevant as a biomarker for response and/or prognosis; its immunological significance, however, remains to be established. Further elucidation of the apparent complex machinery regulating expression of MHC II and its accessory molecules and expression of costimulatory molecules is warranted.

## References

[bib1] Algarra I, Cabrera T, Garrido F (2000) The HLA crossroad in tumor immunology. Hum Immunol 61: 65–73 10658979 10.1016/s0198-8859(99)00156-1

[bib2] Armstrong TD, Clements VK, Martin BK, Ting JP, Ostrand-Rosenberg S (1997) Major histocompatibility complex class II-transfected tumor cells present endogenous antigen and are potent inducers of tumor-specific immunity. Proc Natl Acad Sci USA 94: 6886–6891 9192661 10.1073/pnas.94.13.6886 PMC21254

[bib3] Baskar S, Clements VK, Glimcher LH, Nabavi N, Ostrand-Rosenberg S (1996) Rejection of MHC class II-transfected tumor cells requires induction of tumor-encoded B7-1 and/or B7-2 costimulatory molecules. J Immunol 156: 3821–3827 8621919

[bib4] Becker JC, Brabletz T, Czerny C, Termeer C, Bröcker (1993) Tumor escape mechanisms from immunosurveillance: induction of unresponsiveness in a specific MHC-restricted CD4+ human T cell clone by the autologous MHC class II+ melanoma. Int Immunol 5: 1501–1508 7906140 10.1093/intimm/5.12.1501

[bib5] Blanck G (1999) HLA class II expression in human tumor lines. Microbes Infect 1: 913–918 10614009 10.1016/s1286-4579(99)00226-9

[bib6] Brocke P, Garbi N, Momburg F, Hammerling GJ (2002) HLA-DM, HLA-DO and tapasin: functional similarities and differences. Curr Opin Immunol 14: 22–29 11790529 10.1016/s0952-7915(01)00294-1

[bib7] Bröcker EB, Suter L, Sorg C (1984) HLA-DR antigen expression in primary melanomas of the skin. J Invest Dermatol 82: 244–247 6366074 10.1111/1523-1747.ep12260181

[bib8] Bröcker EB, Zwadlo G, Holzmann B, Macher E, Sorg C (1988) Inflammatory cell infiltrates in human melanoma at different stages of tumor progression. Int J Cancer 41: 562–567 3128489 10.1002/ijc.2910410415

[bib9] Castelli C, Rivoltini L, Mazzocchi A, Parmiani G (1997) T-cell recognition of melanoma antigens and its therapeutic applications. Int J Clin Lab Res 27: 103–110 9266280 10.1007/BF02912443

[bib10] Chen PW, Ullrich SE, Ananthaswamy HN (1994) Presentation of endogenous tumor antigens to CD4+ T lymphocytes by murine melanoma cells transfected with major histocompatibility complex class II genes. J Leukoc Biol 56: 469–474 7930943 10.1002/jlb.56.4.469

[bib11] Clemente CG, Mihm MC, Bufalino R, Zurrida S, Collini P, Cascinelli N (1996) Prognostic value of tumor infiltrating lymphocytes in the vertical growth phase of primary cutaneous melanoma. Cancer 77: 1303–1310 8608507 10.1002/(SICI)1097-0142(19960401)77:7<1303::AID-CNCR12>3.0.CO;2-5

[bib12] Coulie PG, Ikeda H, Baurain JF, Chiari R (1999) Antitumor immunity at work in a melanoma patient. Adv Cancer Res 76: 213–242 10218103 10.1016/s0065-230x(08)60778-2

[bib13] Davis ID (2000) An overview of cancer immunotherapy. Immunol Cell Biol 78: 179–195 10849106 10.1046/j.1440-1711.2000.00906.x

[bib14] Deffrennes V, Vedrenne J, Stolzenberg MC, Piskurich J, Barbieri G, Ting JP, Charron D, Alcaide-Loridan C (2001) Constitutive expression of MHC class II genes in melanoma cell lines results from the transcription of class II transactivator abnormally initiated from its B cell-specific promoter. J Immunol 167: 98–106 11418637 10.4049/jimmunol.167.1.98

[bib15] Goodwin BL, Xi H, Tejiram R, Eason DD, Ghosh N, Wright KL, Nagarajan U, Boss JM, Blanck G (2001) Varying functions of specific major histocompatibility class II transactivator promoter III and IV elements in melanoma cell lines. Cell Growth Differ 12: 327–335 11432807

[bib16] Greenfield EA, Nguyen KA, Kuchroo VK (1998) CD28/B7 costimulation: a review. Crit Rev Immunol 18: 389–418 9784967 10.1615/critrevimmunol.v18.i5.10

[bib17] Guerry D, Alexander MA, Elder DE, Herlyn MF (1987) Interferon-gamma regulates the T cell response to precursor nevi and biologically early melanoma. J Immunol 139: 305–312 3108402

[bib18] Håkansson A, Gustafsson B, Krysander L, Håkansson L (1996) Tumour-infiltrating lymphocytes in metastatic malignant melanoma and response to interferon alpha treatment. Br J Cancer 74: 670–676 8845294 10.1038/bjc.1996.420 PMC2074699

[bib19] Håkansson A, Gustafsson B, Krysander L, Håkansson L (1998) Effect of IFN-alpha on tumor-infiltrating mononuclear cells and regressive changes in metastatic malignant melanoma. J Interferon Cytokine Res 18: 33–39 9475665 10.1089/jir.1998.18.33

[bib20] Håkansson A, Gustafsson B, Krysander L, Hjelmqvist B, Rettrup B, Håkansson L (1999) On down-regulation of the immune response to metastatic malignant melanoma. Cancer Immunol Immunother 48: 253–262 10478642 10.1007/s002620050573 PMC11037119

[bib21] Håkansson A, Gustafsson B, Krysander L, Hjelmqvist B, Rettrup B, Håkansson L (2001) Biochemotherapy of metastatic malignant melanoma. Predictive value of tumour-infiltrating lymphocytes. Br J Cancer 85: 1871–1877 11747328 10.1054/bjoc.2001.2169 PMC2364006

[bib22] Hersey P, Jamal O (1990) Immunohistological relation between DR antigen expression on melanoma cells and infiltration by CD8+ T cells. Pathology 22: 133–139 2147056 10.3109/00313029009063551

[bib23] Kang S, Barnhill RL, Mihm MC, Sober AJ (1993) Histologic regression in malignant melanoma: an interobserver concordance study. J Cutan Pathol 20: 126–129 8320356 10.1111/j.1600-0560.1993.tb00228.x

[bib24] Kosmaczewska A, Ciszak L, Bocko D, Frydecka I (2001) Expression and functional significance of CTLA-4, a negative regulator of T cell activation. Arch Immunol Ther Exp (Warsz) 49: 39–46 11266089

[bib25] Legha SS (1997) The role of interferon alfa in the treatment of metastatic melanoma. Semin Oncol 24: S24–S31 9122731

[bib26] Marelli-Berg FM, Lechler RI (1999) Antigen presentation by parenchymal cells: a route to peripheral tolerance?. Immunol Rev 172: 297–314 10631955 10.1111/j.1600-065x.1999.tb01374.x

[bib27] Marincola FM, Venzon D, White D, Rubin JT, Lotze MT, Simonis TB, Balkissoon J, Rosenberg SA, Parkinson DR (1992) HLA association with response and toxicity in melanoma patients treated with interleukin 2-based immunotherapy. Cancer Res 52: 6561–6566 1423301

[bib28] Martin-Fontecha A, Cavallo F, Bellone M, Heltai S, Iezzi G, Tornaghi P, Nabavi N, Forni G, Dellabona P, Casorati G (1996) Heterogeneous effects of B7-1 and B7-2 in the induction of both protective and therapeutic anti-tumor immunity against different mouse tumors. Eur J Immunol 26: 1851–1859 8765031 10.1002/eji.1830260828

[bib29] Masternak K, Muhlethaler-Mottet A, Villard J, Zufferey M, Steimle V, Reith W (2000) CIITA is a transcriptional coactivator that is recruited to MHC class II promoters by multiple synergistic interactions with an enhanceosome complex. Genes Dev 14: 1156–1166 10809673 PMC316580

[bib30] McGovern VJ (1975) Spontaneous regression of melanoma. Pathology 7: 91–99 1153228 10.3109/00313027509092702

[bib31] Mihm MC, Clemente CG, Cascinelli N (1996) Tumor infiltrating lymphocytes in lymph node melanoma metastases: a histopathologic prognostic indicator and an expression of local immune response. Lab Invest 74: 43–47 8569196

[bib32] Muhlethaler-Mottet A, Otten LA, Steimle V, Mach B (1997) Expression of MHC class II molecules in different cellular and functional compartments is controlled by differential usage of multiple promoters of the transactivator CIITA. EMBO J 16: 2851–2860 9184229 10.1093/emboj/16.10.2851 PMC1169893

[bib33] Mukherji B, Chakraborty NG (1995) Immunobiology and immunotherapy of melanoma. Curr Opin Oncol 7: 175–184 7756383 10.1097/00001622-199503000-00014

[bib34] Nistico P, Tecce R, Giacomini P, Cavallari A, D'Agnano I, Fisher PB, Natali PG (1990) Effect of recombinant human leukocyte, fibroblast, and immune interferons on expression of class I and II major histocompatibility complex and invariant chain in early passage human melanoma cells. Cancer Res 50: 7422–7429 1701342

[bib35] Ostrand-Rosenberg S, Pulaski BA, Clements VK, Qi L, Pipeling MR, Hanyok LA (1999) Cell-based vaccines for the stimulation of immunity to metastatic cancers. Immunol Rev 170: 101–114 10566145 10.1111/j.1600-065x.1999.tb01332.x

[bib36] Pulaski BA, Ostrand-Rosenberg S (1998) Reduction of established spontaneous mammary carcinoma metastases following immunotherapy with major histocompatibility complex class II and B7.1 cell-based tumor vaccines. Cancer Res 58: 1486–1493 9537252

[bib37] Rees RC, Mian S (1999) Selective MHC expression in tumours modulates adaptive and innate antitumour responses. Cancer Immunol Immunother 48: 374–381 10501850 10.1007/s002620050589 PMC11037132

[bib38] Robinson JH, Delvig AA (2002) Diversity in MHC class II antigen presentation. Immunology 105: 252–262 11918686 10.1046/j.0019-2805.2001.01358.x PMC1782669

[bib39] Ruiter DJ, Bhan AK, Harrist TJ, Sober AJ, Mihm Jr MC (1982) Major histocompatibility antigens and mononuclear inflammatory infiltrate in benign nevomelanocytic proliferations and malignant melanoma. J Immunol 129: 2808–2815 6183345

[bib40] Ruiter DJ, Mattijssen V, Broecker EB, Ferrone S (1991) MHC antigens in human melanomas. Semin Cancer Biol 2: 35–45 1912517

[bib41] van Duinen SG, Ruiter DJ, Broecker EB, van der Velde EA, Sorg C, Welvaart K, Ferrone S (1988) Level of HLA antigens in locoregional metastases and clinical course of the disease in patients with melanoma. Cancer Res 48: 1019–1025 3338074

[bib42] van der Stoep N, Biesta P, Quinten E, van den Elsen PJ (2002) Lack of IFN-gamma-mediated induction of the class II transactivator (CIITA) through promoter methylation is predominantly found in developmental tumor cell lines. Int J Cancer 97: 501–507 11802213 10.1002/ijc.1623

[bib43] Yamshchikov G, Thompson L, Ross WG, Galavotti H, Aquila W, Deacon D, Caldwell J, Patterson JW, Hunt DF, Slingluff C-LJ (2001) Analysis of a natural immune response against tumor antigens in a melanoma survivor: lessons applicable to clinical trial evaluations. Clin Cancer Res 7: 909s–916s 11300491

[bib44] Zennadi R, Abdel-Wahab Z, Seigler HF, Darrow TL (2001) Generation of melanoma-specific, cytotoxic CD4(+) T helper 2 cells: requirement of both HLA-DR15 and Fas antigens on melanomas for their lysis by Th2 cells. Cell Immunol 210: 96–105 11520076 10.1006/cimm.2001.1809

